# Modulatory Effects of Fingolimod (FTY720) on the Expression of Sphingolipid Metabolism-Related Genes in an Animal Model of Alzheimer’s Disease

**DOI:** 10.1007/s12035-018-1040-x

**Published:** 2018-04-23

**Authors:** Henryk Jęśko, Przemysław L. Wencel, Walter J. Lukiw, Robert P. Strosznajder

**Affiliations:** 1 Department of Cellular Signalling, Mossakowski Medical Research Centre, Polish Academy of Sciences, 02-106 Warsaw, Poland; 2 Laboratory of Preclinical Research and Environmental Agents, Department of Neurosurgery; Mossakowski Medical Research Centre, Polish Academy of Sciences, 02-106 Warsaw, Poland; 3 LSU Neuroscience Center and Departments of Neurology and Ophthalmology, Louisiana State University School of Medicine, New Orleans, LA 70112 USA

**Keywords:** Alzheimer’s disease, Ceramide, Ceramide-1-phosphate, Fingolimod/FTY720, Kinases, Sphingosine-1-phosphate

## Abstract

Sphingolipid signaling disturbances correlate with Alzheimer’s disease (AD) progression. We examined the influence of FTY720/fingolimod, a sphingosine analog and sphingosine-1-phosphate (S1P) receptor modulator, on the expression of sphingolipid metabolism and signaling genes in a mouse transgenic AD model. Our results demonstrated that AβPP (V717I) transgene led with age to reduced mRNA expression of S1P receptors (*S1PR*s), sphingosine kinase *SPHK2*, ceramide kinase *CERK*, and the anti-apoptotic *Bcl2* in the cerebral cortex and hippocampus, suggesting a pro-apoptotic shift in 12-month old mice. These changes largely emulated alterations we observed in the human sporadic AD hippocampus: reduced *SPHK1*, *SPHK2*, *CERK*, *S1PR1*, and *BCL2*. We observed that the responses to FTY720 treatment were modified by age and notably differed between control (APP^−^) and AD transgenic (APP^+^) animals. AβPP (V717I)-expressing 12-month-old animals reacted to fingolimod with wide changes in the gene expression program in cortex and hippocampus, including increased pro-survival *SPHKs* and *CERK*. Moreover, *BCL2* was elevated by FTY720 in the cortex at all ages (3, 6, 12 months) while in hippocampus this increase was observed at 12 months only. In APP^−^ mice, fingolimod did not induce any significant mRNA changes at 12 months. Our results indicate significant effect of FTY720 on the age-dependent transcription of genes involved in sphingolipid metabolism and pro-survival signaling, suggesting its neuroprotective role in AD animal model.

## Introduction

Alzheimer’s disease (AD) is globally the most widespread age-related neurodegenerative disorder; it undergoes a period of stealthy development and fully manifests itself only after extensive damage has occurred in the central nervous system (CNS). No causal treatment exists despite decades of research effort and the number of cases growing due to population aging. Amyloid β (Aβ) accumulates in the form of extracellular senile plaques and constitutes a hallmark and a central element of AD mechanism, along with the intracellular neurofibrillary tangles of hyperphosphorylated cytoskeletal tau protein [1]. Aβ is released from a transmembrane precursor protein (AβPP) through sequential cleavage by β- and γ-secretase. The V717I “London” mutation of APP has been reported in numerous familial AD (FAD)/early-onset AD families [2, 3]. The change occurs near the γ cleavage site; it increases Aβ production and slightly modifies the γ-secretase cleavage point, shifting the proportions in favor of Aβ_42_ [4], the isoform that correlates with high neurotoxicity and with early onset of the pathology [5]. Some groups, however, observed that V717I mutation might also alter processing by β-secretase [4]. The resulting increased production of Aβ has also been found in vitro to impact the levels of tau protein, potentially further enhancing the pathology [4]. Synergy has also been reported between Aβ_42_ toxicity and ApoE allele [6]. Moreover, the effect of “London” mutation leads to different sets of symptoms in patients of various ethnic origins and has been suggested to be modified by other genetic or environmental factors [2]. Mouse model expressing the V717I AβPP under control of the neuron-specific mouse *thy-1* promoter (AβPP expression strongest in large pyramidal neurons of hippocampus, cerebral cortex, and amygdala) has been created and characterized [7]. Although the limited set of re-created traits is a general problem in AD modeling, the AβPP V717I mice recapitulate a number histochemical, behavioral, electrophysiological, and biochemical features of the disease [7, 8]. Importantly, the protracted course of AD pathogenesis is reflected in the gradual appearance of Aβ deposits enriched in the Aβ_42_ isoform; these deposits frequently formed near acetylcholinesterase-immunoreactive structures and were confirmed to attract and activate astrocytes and microglia [7–9]. Interestingly, the timing of the observed phenotypical disturbances (decreased synaptic plasticity, disturbed glutamatergic signaling, cognitive impairment, aggression, etc.) appears to precede large-scale Aβ/plaque deposition, pointing to the necessity of identification of the early, low-intensity changes that lead to the later devastating outcome [7].

The vast potential importance of sphingolipid metabolism in AD stems both from their roles in the regulation of cellular survival and from their structural roles in lipid rafts. Sphingosine-1-phosphate (S1P) produced by sphingosine kinases (SphK1 and SphK2) is a central element of a signaling network that influences cell survival, proliferation, differentiation, and mature neuron phenotype (neurite morphology, neurotransmitter secretion, and synaptic plasticity). S1P may act either as an intracellular second messenger, or through cell surface G-protein-coupled receptors S1PR1 to S1PR5. Sphingolipid signaling appears to be engaged in bi-directional interactions with Aβ and its precursor protein. Aβ can modulate both the expression and activities of sphingolipid-metabolizing enzymes and S1P receptors in cellular models [10] and AD cases [11]. Genes upregulated in AD included ceramide synthases CERS1 (also termed *LASS1*, mammalian homolog of yeast longevity-assurance gene 1) and CERS2 (*LASS2*), S1P lyase (*SGPL1*), or serine palmitoyltransferase (*SPTLC2*), while the acid ceramidase (*ASAH1*) and ceramide kinase (*CERK*) were reduced [12]. The changes in gene expression correspond well with the observed reduction of brain levels of S1P. S1P has strong anti-apoptotic activity in most cell types, and its loss correlates in AD brains with the extent of degenerative changes in the structures affected [13]. The change occurs very early in AD, potentially hinting at the engagement of sphingolipid signaling relatively upstream in the elusive cascade that drives the pathology [13]. The accompanying upregulation of selected ceramide species may not only signal apoptosis, but also affect the regulation of β- and γ-secretases and AβPP processing [12, 14–16]. The influence of sphingolipids may be largely mediated through their role in plasma membrane microdomains called lipid rafts [17], which display extensive links with AβPP metabolism, Aβ production, and aggregation [18, 19]. Moreover, S1PRs modulate neuron-microglia interactions, microglial activation, and secretion of neurotoxic compounds and seem to influence the fine balance between the restorative and destructive outcomes of astrogliosis [20, 21].

**Fig. 1:**
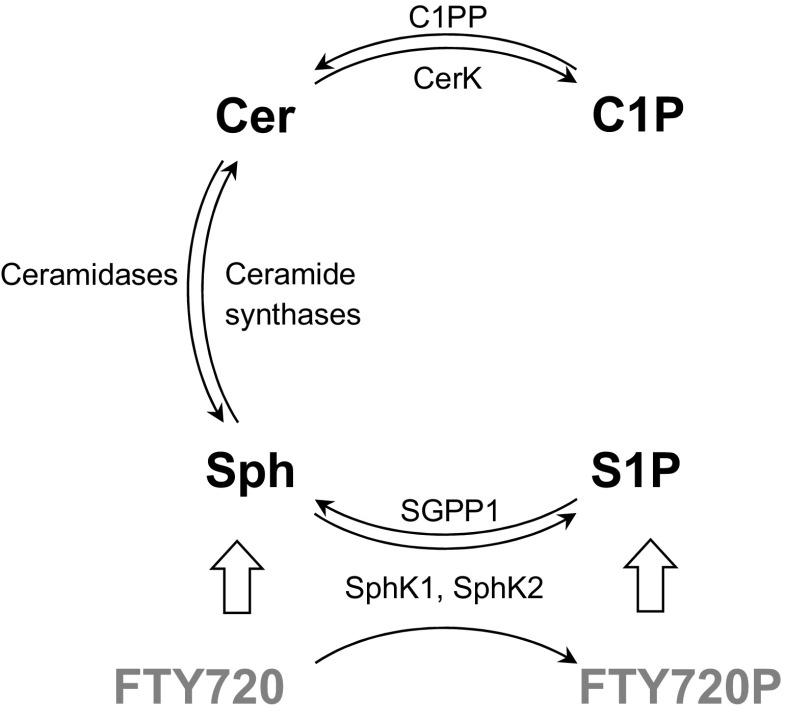
Metabolic relationship between ceramide and sphingosine-1-phosphate. Ceramide (Cer) serves as a substrate for ceramidase which produces sphingosine (Sph). Sphingosine can be phosphorylated to sphingosine-1-phosphate (S1P) by either of the two sphingosine kinases SphKs, while ceramide is phosphorylated to ceramide-1-phosphate (C1P) by the ceramide kinase CerK. The reactions are reversed by S1P and C1P phosphatases (SGPP1 and C1PP, respectively). The thick arrows point to the biological activities of FTY720, which is a structural analogue of sphingosine; in the target tissue, FTY720 is phosphorylated by SphKs to become FTY720P, an S1P analogue and S1PR ligand

For years, the roles of sphingolipids in the cellular survival was described using the *sphingolipid rheostat* model where the apoptosis-activating influence of ceramide and sphingosine is offset by the pro-survival signaling of their closely linked metabolites S1P and ceramide-1-phosphate (C1P) [22]. Ceramide can be converted to sphingosine by ceramidases; an opposite reaction is catalyzed by ceramide synthase (Fig. 1) and is one of several known pathways of ceramide generation in the cell. The roles ascribed to the bioactive sphingolipids in apoptosis/survival signaling still largely stand. C1P is produced from ceramide by the calcium-sensitive CERK (Fig. 1). C1P has been shown to exert negative influence on ceramide generation by acid sphingomyelinase [23] and serine palmitoyltransferase [24], thus blocking apoptosis. C1P also modulates cell proliferation and inflammatory/migratory phenotypes [25]. The existence of a specific cell surface C1P receptor has been postulated [26]. The cellular activities of C1P are known to be mediated by, e.g., PI3 kinase, Akt/protein kinase B (PKB), phospholipase A2, protein kinase C, NF-κB, iNOS-produced nitric oxide, and ROS [27–29]. S1P, a product of sphingosine phosphorylation (Fig. 1), in most cases exerts pro-survival influence via the Akt and extracellular signal-regulated kinase (ERK) pathways, increasing the expression of Bcl-2 and reducing the levels of Bax and the apoptosis activator protein harakiri (Hrk) [30]. However, prolonged accumulation of S1P may lead to neurodegeneration via calpain, endoplasmic reticulum stress, and cyclin-dependent kinase 5 [31]. In turn, some ceramide species generated by CerS2 may actually exert anti-apoptotic influence in HeLa cell model [32]. In addition to the sometimes contradictory activities of various closely related compounds, the ever-expanding network of feedbacks and fine-tuning dependencies between the enzymes, receptors, and sphingolipid species must be taken into account. Importantly, sphingolipids and small-molecule enzyme/receptor modulators can influence regulatory pathways in the nucleus. S1PRs signal through G_q_, G_12/13_, and G_i_ proteins, which in turn modulate the PI3 kinase-Akt pathway, ERK, c-Jun N-terminal kinase (Jnk), phospholipase C, or adenylate cyclase. These pathways relay their signals, e.g., to transcription factors such as the activator protein AP-1, or nuclear factor κB (NF-κB). Some relevant examples of S1PR influence on gene regulation include the feedback impact of S1PR2 activation on SphK1 expression, or induction of the cyclooxygenase COX-2 by S1PR3 (which can lead to free radical buildup)—both occur via AP-1 [33–35]. Importantly, *CERS4* and *CERS5* genes are also regulated by AP-1 [36]. The link between S1PR activation and gene expression is also significant for the analysis of the effects of FTY720/fingolimod, a Food and Drug Administration- and European Medicines Agency-approved orally bioavailable drug for the treatment of relapsing remitting multiple sclerosis [37]. The current therapeutic usage of fingolimod is based on its immunomodulatory activity. FTY720 is a structural sphingosine mimetic drug capable of penetrating the blood-brain barrier [37]. FTY720 becomes phosphorylated in the tissue and becomes an S1P analogue, gaining the ability to bind S1PRs (with the disputed exception of S1PR2 [21]), activating them and exerting other effects typical for S1P, such as receptor internalization. FTY720 is able to induce a gene expression program in neurons that modifies their phenotype and potentially might mitigate the loss in connectivity observed in the course of neurodegenerative disorders [38]. FTY720 also causes expression and secretion of a set of neurotrophic factors by astrocytes [39]. The S1PR1-mediated effects of fingolimod have already been successfully tested in vivo in two mouse models of Parkinsonian neurodegeneration [40]. Additionally, both S1P and FTY720P have also been found to bind and inhibit class I histone deacetylases (HDACs) in the nucleus [41, 42]. Class I HDACs modulate synaptic plasticity-linked gene expression, may negatively affect cognitive functions, but can be important for neurogenesis and DNA repair [43]. All the effects exerted by FTY720 / FTY720P must be understood and taken into account before the potential pro-survival influence of the compound can be exploited in potential AD therapies.

## Aim

Our study focused on the modulatory effect of fingolimod (FTY720) on the age-dependent gene expression profile of enzymes and receptors engaged in S1P/ceramide signaling and anti-apoptotic Bcl-2 protein in brain parts of transgenic AD mice.

## Materials and Methods

### Animal Housing and Treatment

Female FVB-Tg(Thy1; APP LD2/B6) mice, aged 3, 6, or 12 months, were used. The animals overexpressed human AβPP with the “London” V717I mutation under control of a fragment of Thy1 promoter with specificity towards brain and spinal cord neurons (APP^+^). Mice that did not inherit the transgene were used as controls (APP^−^).

Mice were bred under specific pathogen-free (SPF) conditions by the Animal House of the Mossakowski Medical Research Centre PAS, Warsaw, Poland. The mice were housed in controlled temperature and humidity conditions and 12-h light/dark cycle. Animals were treated for 2 weeks daily with FTY720, a sphingosine analog and S1P receptor modulator, or the appropriate vehicle (controls).

The doses used were based on analysis of data from earlier studies [44–46]. Animals were weighed to assess correct injection volume/dose. FTY720 was dissolved in 0.9% NaCl, diluted, and administered intraperitoneally for 2 weeks daily (15 injections) in a dose of 1 mg/kg b.w.; controls received NaCl only. One day after the last treatment, animals were anesthetized and decapitated, and cerebral cortices were isolated on ice and flash-frozen in liquid nitrogen.

Every effort has been made to minimize the number of animals used and reduce the amount of pain, distress, and/or discomfort. All experiments were approved by the IV Local Ethics Committee for Animal Experimentation in Warsaw and were carried out in accordance with the EC Council Directive of November 24, 1986 (86/609/EEC), following the ARRIVE guidelines and guidelines published in the NIH Guide for the Care and Use of Laboratory Animals and the principles presented in the “Guidelines for the Use of Animals in Neuroscience Research” by the Society for Neuroscience.

### Gene Expression Measurement—Real-Time Polymerase Chain Reaction

Brain cerebral cortices and hippocampi were isolated on ice and flash-frozen in liquid nitrogen. RNA was extracted using Chomczynski method with TRI-reagent according to manufacturer’s protocols (Sigma-Aldrich/Merck). DNA was digested with DNase I (Sigma-Aldrich). The concentration and purity of RNA were assessed spectrophotometrically (A_260_/A_280_ method). Reverse transcription of 4 μg of total RNA was performed with avian myeloblastosis virus (AMV) reverse transcriptase and random primers (High Capacity Reverse Transcription Kit, Applied Biosystems, Foster City, CA, USA). Real-time PCR was performed with TaqMan Gene Expression Assay kits using ABI PRISM 7500 machine (both reagents and equipment—Applied Biosystems). Each sample was analyzed in tri- to quadruplicate. Gene expression was calculated using the ddCt method and normalized against actin B (ACTB).

### Microarray Measurement of Gene Expression Using DNA Arrays

Total DNA, RNA, and proteins were isolated from control and AD-affected human brains in primary culture using TRIzol (Invitrogen) as previously described by our laboratory [47–51]; RNA quality was assessed using an Agilent Bioanalyzer 2100 (Lucent Technologies/Caliper Technologies, Palo Alto, CA, USA) and RNA integrity number (RIN) values were typically 8.0–9.0 indicating high-quality total RNA [50–54]. Control and AD brain RNA samples were labeled and hybridized and analyzed using GeneChips (Affymetrix, Palo Alto CA, USA) as previously described in detail by our group [49–54].

### Statistical Analysis

mRNA expression levels (Rq) represent mean values ± S.E.M from 2 to 6 independent experiments carried out in triplicate to quadruplicate. Statistical analysis was performed using two-way analysis of variance (ANOVA) with Tukey post hoc test in GraphPad Prism (GraphPad Software, San Diego, CA). Statistical significance was accepted at *p* < 0.05.

## Results

**Fig. 2:**
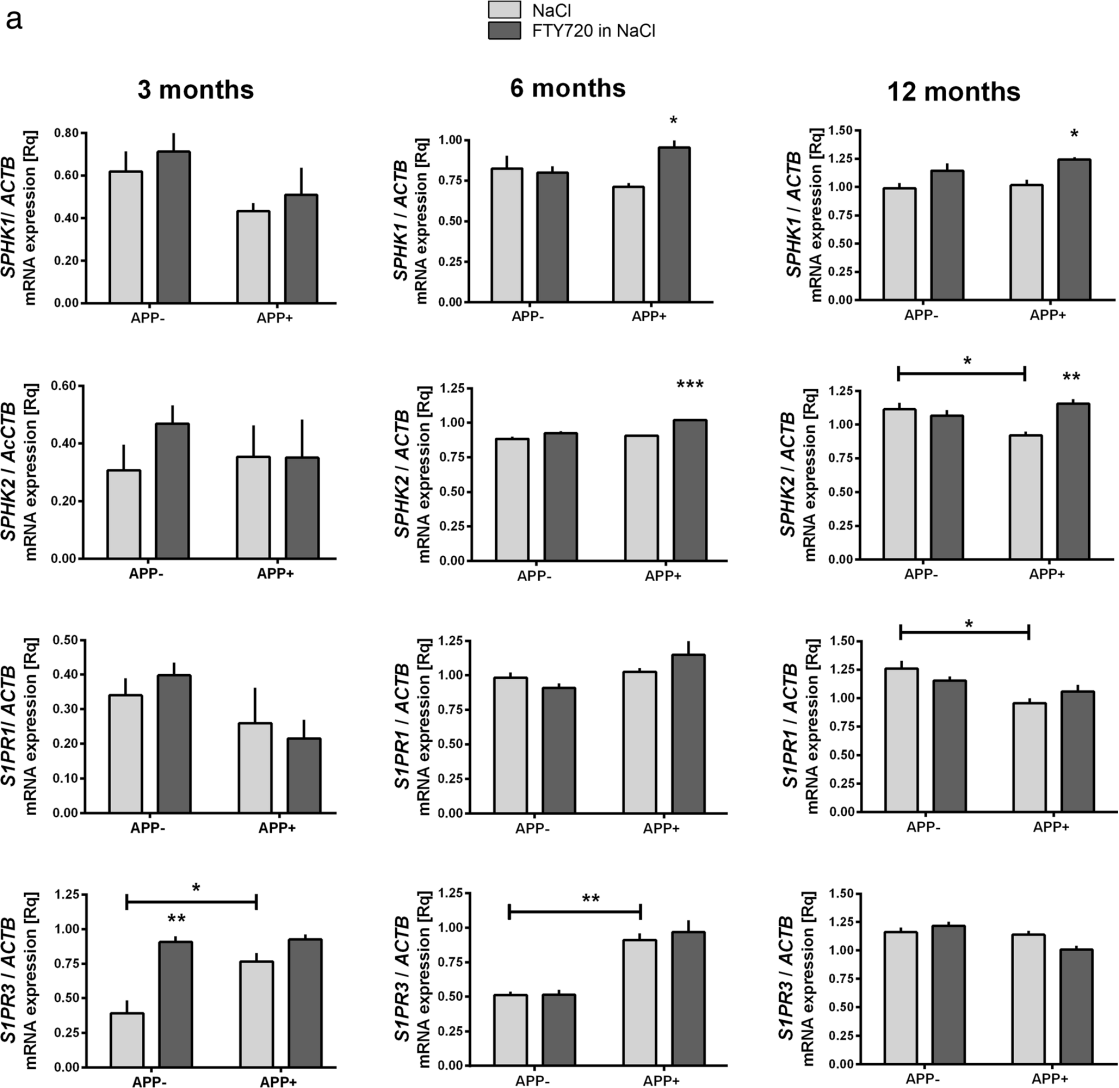

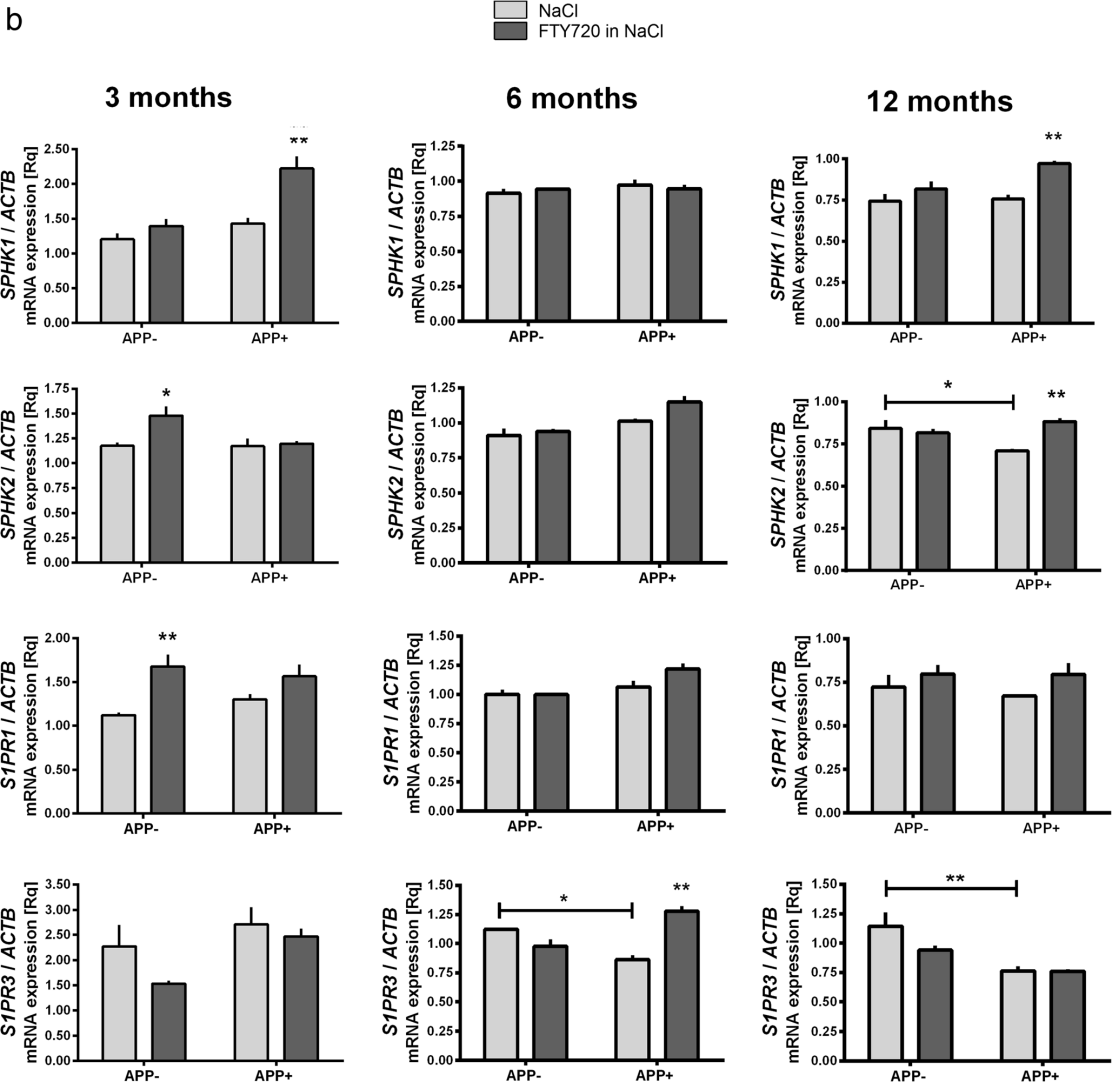
The influence of AβPP V717I expression and administration of FTY720 on the expression of sphingosine kinases and S1P receptors in the mouse brain. Changes in the mRNA levels of enzymes regulating S1P metabolism and signaling measured using real-time PCR in the brain cortex (A) and hippocampus (B) of 3-, 6-, and 12-month-old APP-transgenic and control mice. *p < 0.05; **p < 0.01; ***p < 0.001 as compared to the appropriate controls; ANOVA with Tukey post hoc test

**Fig. 3:**
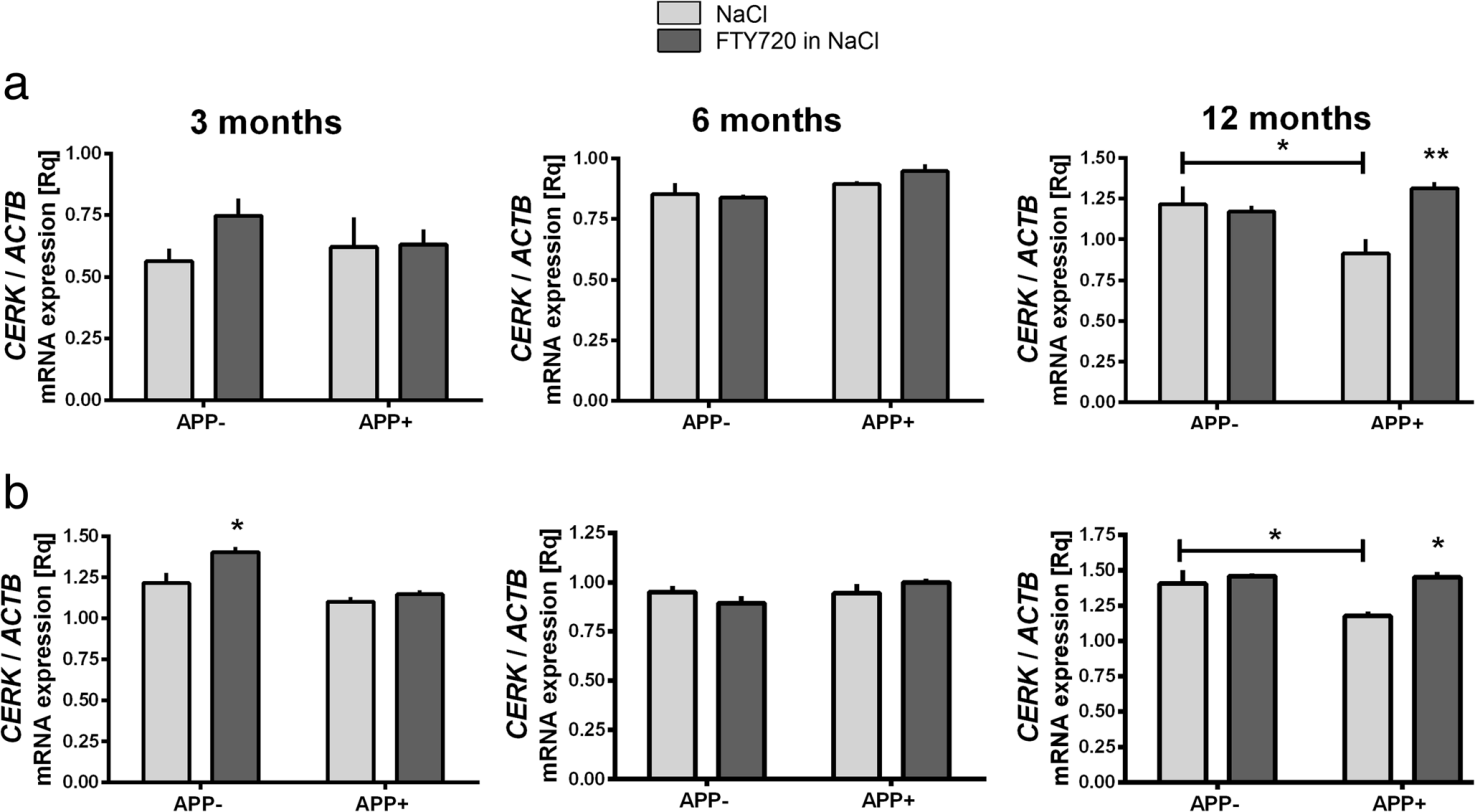
The influence of AβPP V717I expression and administration of FTY720 on the expression of ceramide kinase in the mouse brain. Changes in the mRNA levels of ceramide kinase measured using real-time PCR in the brain cortex (A) and hippocampus (B) of 3-, 6-, and 12-month-old APP-transgenic and control mice. *p < 0.05; **p < 0.01 as compared to the appropriate controls; ANOVA with Tukey post hoc test

**Fig. 4:**
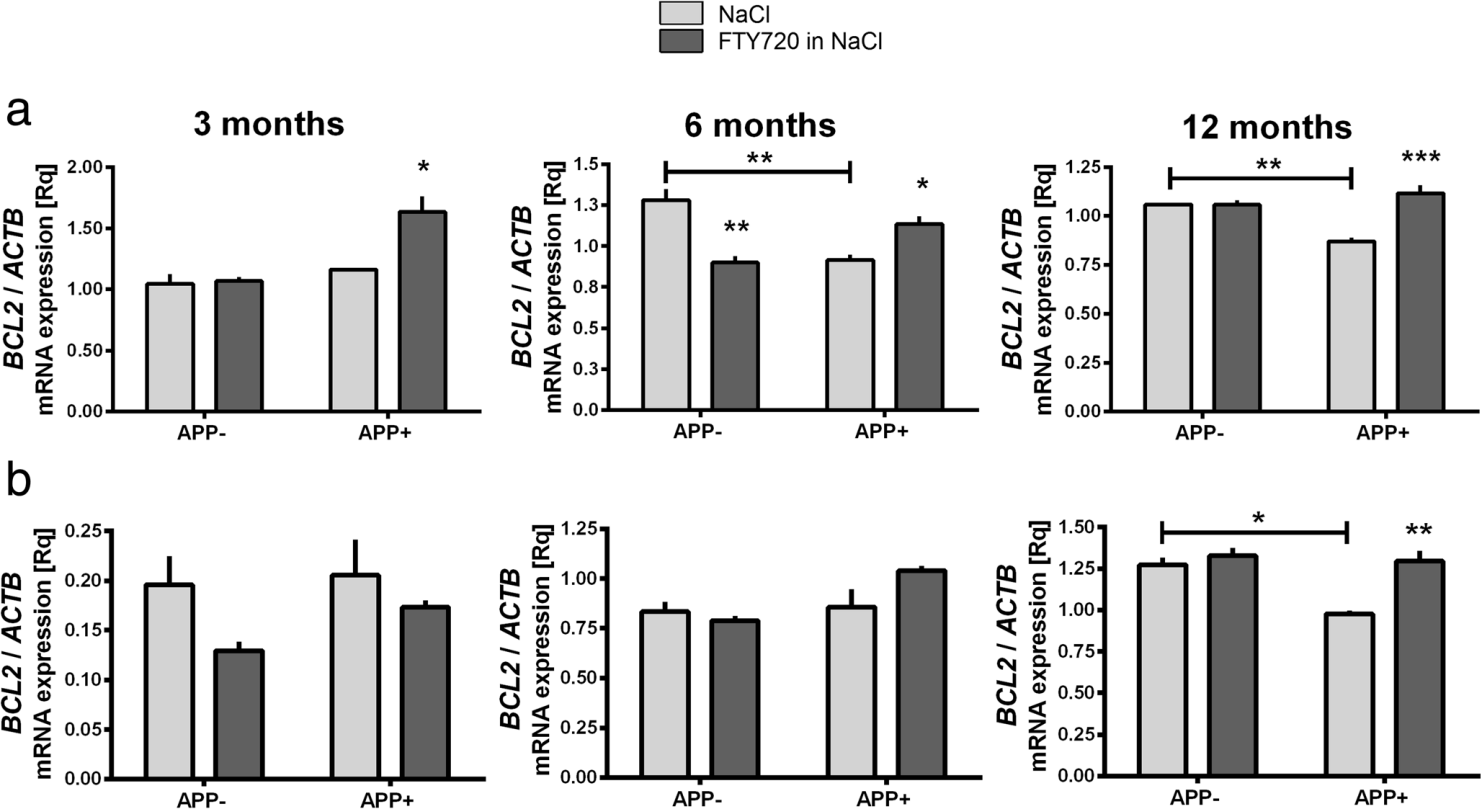
The influence of AβPP V717I expression and administration of FTY720 on the expression of the anti-apoptotic *BCL2* in the mouse brain. Changes in the mRNA levels of *BCL2* measured using real-time PCR in the brain cortex (A) and hippocampus (B) of 3-, 6-, and 12-month-old APP-transgenic and control mice. * *p* < 0.05; ** *p* < 0.01; *** *p* < 0.001; as compared to control; ANOVA with Tukey post hoc test

Significant alterations in the levels of ceramide(s) and S1P have been observed in human AD brains and in animal models [12]. To characterize the underlying alterations in gene expression, we analyzed the effect of AβPP V717I transgene on the levels of mRNAs linked to sphingolipid metabolism and apoptosis in the cerebral cortex and hippocampus of 3-, 6-, and 12-month-old mice. We also evaluated the influence of mutant AβPP on the effects exerted by fingolimod (FTY720), a sphingosine analog and S1P receptor modulator. A number of genes whose expression responded to V717I AβPP/FTY720 were included in Figs. 2, 3, and 4.

### mRNA Expression Levels of Enzymes Engaged in S1P Metabolism and of S1P Receptors

SphKs are crucial enzymes of the *sphingolipid rheostat*; their product S1P can signal through cell surface receptors (S1P1 to S1P5), or as an intracellular second messenger. Therefore, we analyzed the effect of AβPP V717I on the mRNA levels of sphingosine kinases and S1P receptors. As demonstrated in Fig. 2(A), the presence of the AβPP transgene has led in 12-month-old brain cortex to reduced expression of the sphingosine kinase *SPHK2*. The crucial, best-characterized S1P receptor *S1PR1* was also lower in APP^+^ cortex at the age of 12 months, while *S1PR3* was elevated in the cortex at 3 and 6 months (Fig. 2(A)). We also noted an apparent reduction in the levels of *SPHK1* and *S1PR1* mRNAs in the 3 months old cortex, although it did not reach significance. In the hippocampus, *SPHK2* was again lower in APP^+^ mice at 12 months (Fig. 2(B)). In contrast to the cortex, only *S1PR3* was changed—we found it reduced in APP^+^ samples at 6 and 12 months of age (Fig. 2(B)). These differential changes of S1P receptors in response to AβPP (V717I) expression might signal an important difference between the brain parts and have vast significance for the sphingolipid-controlled survival and function of brain neurons.

FTY720, a structural analog of sphingosine, is a substrate for endogenous SphKs. The resulting FTY720 phosphate mimics the activity of S1P, including binding to the S1P receptors. Like numerous cell surface receptor agonists, FTY720P can lead to S1PR internalization, effectively modulating their protein level at the cell surface. However, FTY720 / FTY720P can exert a number of other effects including changes in neuronal gene expression [38], as demonstrated by a previous study on a rat AD model induced by single Aβ injection [55]. In our hands, the long-term presence of AβPP (V717I) has notably modified the cortical responses to the treatment with FTY720. mRNA expression of both sphingosine kinases was elevated after FTY720 treatment, only in 6- and 12-month-old APP^+^ brain cortex (Fig. 2(A)); Fingolimod also increased *SPHK1* mRNA in the 3- and 12-month-old APP^+^ hippocampus and *SPHK2*, only at 12 months (Fig. 2(B)). No changes in S1P receptor expression were noted in the APP^+^ cortex (Fig. 2(A)), while in the hippocampus *S1PR3* was upregulated in response to FTY720 only at the age of 6 months (Fig. 2(B)). In turn, the control animals responded to FTY720 with increased expression of *S1PR3* in the cortex at the age of 3 months, and with increased expression of *SPHK2* and *S1PR1*—in 3 months old hippocampus. This suggests a potentially pro-survival activity of fingolimod also in the control brain, although the observed changes are much less numerous, and disappear in older age.

### mRNA Level of Ceramide Kinase

Our data demonstrated that the presence of the V717I AβPP transgene has led to a reduction of the ceramide kinase *CERK* in the brain cortex (Fig. 3(A)) and hippocampus (Fig. 3(B)) of 12-month-old mice. In APP^+^ animals, FTY720 treatment has led to elevation of *CERK* mRNA at the age of 12 months in both tissues. The change might have some adaptive value in the presence of Aβ, given the well-known role of C1P in cell survival.

### mRNA Expression of the Apoptosis Regulator *BCL2*

The alteration of S1P-ceramide balance in favor of ceramide accumulation may lead to strong pro-apoptotic signal. We have investigated gene expression of the potent anti-apoptotic protein Bcl-2 in response to the presence of V717I AβPP transgene and FTY720 treatment. Our result demonstrate that AβPP expression has reduced the levels of *BCL2* mRNA in 6- and 12-month-old brain cortex (Fig. 4(A)) and only in 12-month-old hippocampus (Fig. 4(B)). AβPP has also modified the response of apoptotic regulatory signaling to FTY720. Fingolimod elevated *BCL2* mRNA in APP^+^ cortex at the age of 3, 6, and 12 months (Fig. 4(A)), while in the hippocampus the elevation was observed only at 12 months (Fig. 4(B)). In contrast, in APP^−^ mice FTY720 has caused a reduction in the cortical *BCL2* expression at the age of 6 months.

The influence of AβPP on the levels of mRNAs linked to sphingolipid metabolism was compared to results obtained in human hippocampus of sporadic AD cases.

### DNA Array Studies in Human Hippocampal CA1

**Fig. 5:**
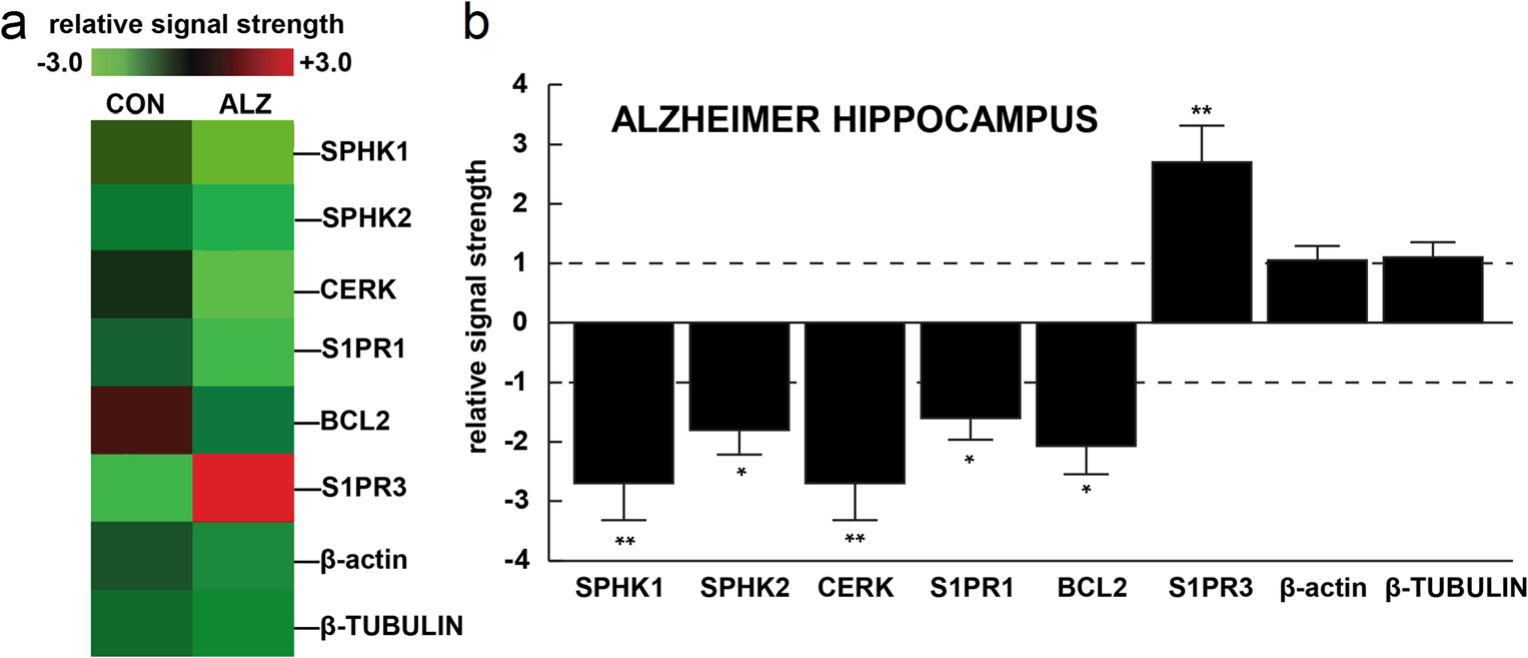
Changes in hippocampal expression of sphingosine kinases, *CERK*, S1P receptors, and *BCL2* in human sporadic AD hippocampus. (a) Using a gene expression mRNA array analytical approach reduced SPHK1, SPHK2, CERK, S1PR1, and BCL2 and an increase in S1PR3 was found in sporadic AD brains (marked ALZ) compared to age-matched controls (CON); no changes were found in the control biomarkers β-actin or β-TUBULIN in the same samples; (b) mean results quantified in bar graph format. RNAs were isolated from samples of hippocampal CA1 of sporadic AD cases (PMI of 3 h or less), all RNA integrity (RIN) numbers were between 8.0 and 9.0, clinical dementia ratings (CDR) for all ALZ patients were between 1.0 and 3.0 (mild to severe dementia), and CDR for controls (CON) ranged between 0.0 and 0.5, [56–58]; all CON or ALZ samples were subjected to DNA array (GeneChip) analysis as described in Materials and Methods. A dashed black horizontal line has been placed at +1 (upregulated gene expression) and − 1 (downregulated gene expression) for ease of comparison. *p < 0.05; **p < 0.01 as compared to the appropriate controls; ANOVA with Tukey post hoc test

Global gene expression patterns in the human hippocampal CA1 region of control versus age-matched sporadic Alzheimer’s disease (sAD) brain gave comparable results to the studies in FVB mice. sAD brains showed a similar reduction in *SPHK1*, *SPHK2*, *CERK*, *S1PR1*, and *BCL2* and an increase in *S1PR3* while the control markers β-actin and α-tubulin showed no significant change (Fig. 5).

## Discussion

The FAD/early-onset AD-linked V717I “London” AβPP mutation [2, 3] increases AβPP cleavage into Aβ and shifts the proportions of the cleavage products in favor of the highly neurotoxic Aβ_42_ [4]. Little is known on other aspects of the influence of the mutation on the (still elusive) functions of AβPP in neurons. The mouse model used in this study expresses V717I AβPP under the control of a neuron-specific promoter [7]. It recapitulates a relatively broad set of AD-linked histochemical, behavioral, electrophysiological, and biochemical features appearing in an age-dependent sequence [7, 8]. The order of appearance of various aspects of pathology in V717I mice has confirmed the limitedness of our current mechanistic knowledge on the crucial early phase of the disease [7].

We observed that the expression of AβPP V717I transgene induced relatively few but important changes in the levels of sphingolipid metabolism genes. Most of these alterations took place in the aging brain (12 months); significant Aβ-related pathology is observed in V717I AβPP mice about the age of 11–12 months and beyond, and in our model the shift in sphingolipid signaling appears to co-occur with these changes (Figs. 2 and 3) [7]. Twelve-month-old mouse cortex displayed reduction of *SPHK2* and *S1PR1* mRNAs. In the hippocampus, we again observed reduction of *SPHK2* at 12 months of age, but only *S1PR3* was downregulated among the receptors (at 6 and 12 months)—Fig. 2. This is much in accordance with the published tendency towards a shift from S1P synthesis and signaling to ceramide generation [11, 13]. The change in ceramide kinase at 12 months of age in both brain parts (Fig. 3) is also in agreement with the published trends in disease models and actual AD cases [11–13]. The downregulation of SPHK1, *SPHK2*, and *CERK* was also evident in the human CA1 area of sAD cases. However, the detailed pattern of changes in the mouse AβPP model is slightly different from our AD results (as *S1PR1* mRNA is reduced and S1PR3 elevated in human CA1—Fig. 5) and from that reported in some other human works [12], clearly reflecting the heterogeneity of the disease pathogenesis (mainly FAD/early-onset vs. sporadic AD) and species-specific differences. The ambiguous role of *SPHK2* in apoptosis [59] means that the observed changes might have varied outcome, depending on subtle changes in the kinase subcellular localization, levels and activities of other signaling proteins, lipids, etc.

Bcl-2 (B cell lymphoma 2) is a prototype of a family of apoptosis-regulating proteins that react to cellular stress (free radical/chemical damage, growth factor deprivation, or cytoskeletal abnormalities). Significant redundancy ensures the precision and safety of the operation of most of the constituents of the pathway, with the exception of Bcl-2 which alone is able to ensure cellular survival [60]. Lower mRNA for the anti-apoptotic *BCL2* we have consistently observed in the human AD CA1 area, in 12-month-old hippocampus, and in the brain cortex of 6- and 12-month-old mice (Figs. 4 and 5) might alter the balance of cell survival signaling, in agreement with the repeatedly demonstrated role of Bcl-2 in animal models of AD-type neurodegeneration induced by either genetic manipulation or neurotoxic insults [61, 62]. In addition, Bcl-2 protein levels may be further reduced by upregulated translation inhibitor miR-34a, as observed in a mouse genetic AD model [63].

Behavioral alterations in AβPP V717I transgenic mice start earlier than measurable disturbances in Aβ levels, and finding their molecular/biochemical correlates may hint at the upstream processes that disturb neuronal function long before clear symptoms are recognizable [7]. Interestingly, we have observed a temporary rise in the cortical mRNA levels of sphingosine-1-phosphate receptor *S1PR3* at the age of 3 and 6 months (Fig. 2(A)). Mechanistically, this is to some degree in contrast with the available findings from the immune system where Aβ selectively targeted the expression of *S1PR2* and *S1PR5* but not *S1PR3* [10]. The temporary upregulation of receptor expression in younger animals might have adaptive value for cells exposed to the influence of mutant AβPP/Aβ.

Pre-clinical research on FTY720 in neurodegenerative disorders includes AD, Parkinson’s disease, or Huntington’s disease. Although primarily used as the S1P receptor agonist, in some circumstances the antagonistic effects of FTY720P-induced S1PR internalization may be more pronounced [21]. This phenomenon is exploited in its therapeutic applications thus far, but makes interpretation of the results of its experimental administration much more difficult. Chronic, peripheral FTY720 treatment has been shown to attenuate histological damage and reduce behavioral deficits in the hippocampal CA1 field of Aβ_42_-injected rats [64]; in vitro FTY720 is able to reduce Aβ production by primary mouse neurons, although with some increase of the Aβ_42_/Aβ_40_ ratio [65]. The mechanism of fingolimod’s action may include effects on apoptotic signaling (as an S1P analog, it may counteract the effects of ceramide), or on AβPP/Aβ metabolism. S1P is known to modulate the secretion of numerous neurotransmitters, growth factors, or hormones [66] and it can also change the cellular levels and secretion of AβPP [67]. SphK overexpression or glycosphingolipid/ceramide depletion inhibits AβPP maturation, its transport, and Aβ toxicity while ceramide stabilizes β-secretase and increases Aβ production [14, 15, 68]. The matters still seem to be far from clear, as other works show that also S1P can modulate BACE1 [69]. Moreover, the idea of the clear-cut S1P vs. ceramide antagonism is being criticized for skipping numerous aspects of sphingolipid signaling, such as S1P neurotoxicity [31, 70].

We observe widespread and age-dependent differences in the sensitivity of AβPP-expressing and control brain to FTY720. The expression of sphingosine kinases, *CERK*, and *BCL2* was upregulated by FTY720 in the APP^+^ brain. The complex interactions of sphingolipids with nuclear gene regulation suggest close links between these pathways, but detailed understanding is missing. The ability of the sphingosine mimetic drug FTY720/fingolimod to modulate gene expression in neurons and astrocytes [38, 39] is probably due in large part to the links observed between S1P receptor signaling and the activities of crucial transcription factors including NF-κB, Yin-Yang-1, or Notch [71–75]. However, the inhibition of class I HDACs by FTY720 / FTY720P must also be considered a potential mechanism [41, 42]. Literature data shows FTY720 being able to ameliorate the Aβ-induced changes in the expression of crucial genes, including caspase-3, nuclear factor κB, brain-derived neurotrophic factor, tumor necrosis factor α, interleukin 1β, or mitogen-activated protein kinases [55, 76]. Our results contribute to the knowledge on the influence of S1P and its mimetic FTY720P on the transcription of genes controlling relevant cellular processes. The topic must be characterized in depth to enable successful and predictable application of fingolimod in AD—either as a research tool or (potentially) as a therapeutic compound.
